# Role of opioid system in verapamil-induced antinociception in a rat model of orofacial pain

**Published:** 2014

**Authors:** Esmaeal Tamaddonfard, Amir Erfanparast, Mina Taati, Milad Dabbaghi

**Affiliations:** 1 *Department of Basic Sciences, Faculty of Veterinary Medicine, Urmia University, Urmia, Iran;*; 2 *DVM student, Faculty of Veterinary Medicine, Urmia University, Urmia, Iran.*

**Keywords:** Formalin, Morphine, Naloxone, Orofacial pain, Verapamil

## Abstract

Calcium, through its various channels involves in local, spinal and supra-spinal transmission of pain. In the present study, we investigated the separate and combined treatment effects of verapamil (a calcium channel blocker), morphine (an opioid agonist) and naloxone (an opioid antagonist) on pain in the orofacial region of rats. Orofacial pain was induced by subcutaneous (SC) injection of formalin (50 µL, 1.5%) into the left upper lip side, and the time durations spent face rubbing with epsilateral forepaw were recorded in three min blocks for a period of 45 min. Formalin induced a biphasic pattern (first phase: 0-3 min; second phase: 15-33 min) of pain. Intraperitoneal (IP) injections of verapamil (2 and 8 mg kg^-1^) and morphine (2 and 4 mg kg^-1^) suppressed orofacial pain. Co-administration of sub-analgesic doses of verapamil (0.5 mg kg^-1^) and morphine (1 mg kg^-1^) produced second phase analgesia. Both phases of formalin-induced pain were suppressed when an analgesic dose (2 mg kg^-1^) of verapamil co-administered with a sub-analgesic dose (1 mg kg^-1^) of morphine. The SC injection of naloxone (2 mg kg^-1^) alone with no effect on pain intensity, prevented the antinociceptive effects induced by morphine (2 mg kg^-1^), but not verapamil (2 mg kg^-1^). The obtained results showed antinociceptive effects for verapamli and morphine on orofacial pain. Co-administrations of verapamil and morphine produced antinociceptive effects. It seems that opioid analgesic system may not have a role in the verapamil-induced antinociception.

## Introduction

Calcium ions (Ca^2+^) serve as important mediators of cell signaling in both excitable and non-excitable cells. Elevation in intracellular Ca^2+^ levels triggers physiological responses that include muscle contraction, hormone secretion, neurotransmission, activation of Ca^2+^-dependent enzymes and Ca^2+^-dependent gene transcription.^1^ Calcium can enter cells by several classes of channels including voltage-, store-, second messenger-, and receptor-gated Ca^2+^ channels.^2^ The voltage-gated calcium channels (VGCCs) can be divided based on their structural similarities of the channel-forming α-subunit (Ca_v_1, Ca_v_2 and Ca_v_3), or their sensitivity to blockade by pharmacological agents (L, N, P/Q, R and T-type). Four different isoforms exist for L-type calcium channels (LTCCs) including Ca_v_1.1, Ca_v_1.2, Ca_v_1.3 and Ca_v_1.4.^3^

The LTCCs are widely distributed in the nervous system,^4^ and are involved in local, spinal and supra-spinal transduction of pain.^5^^-^^7^ At the level of nociceptors, LTCCs contributed to release of calcitonin gene related peptide (CGRP).^8^ Nimodipine, a LTCC blocker, decreased the expression of CGRP in the trigeminal nucleus caudalis.^9^ Modulation of LTCCs altered the firing modes of thalamocortical neurons and pain responses.^10^ The LTCCs are also involved in morphine-induced analgesia and chronic tolerance. The LTCC blockers including verapamil, diltiazem, nifedipine, nimodipine, nicardipine, flunarizine and cinnarizine have extensively used for treatment of various models of pain.^6^

The orofacial region is one of the most densely innervated (by the trigeminal nerves) areas of the body, which focuses some of the most common acute, chronic and referred pains.^11^ The orofacial formalin test was introduced and completed by Clavelou *et al*. ^12^^,^^13^ This model of orofacial pain has been frequently used with success in the study of the pain originating from orofacial region.^14^^-^^17^

In the present study, we investigated the effects of verapamil, a LTCC blocker, on formalin-induced orofacial pain in rats. Due to the involvement of LTCCs in morphine-induced analgesia,^6^ co-administration of verapamil and morphine was also examined. Naloxone pretreatment was performed to clarify the involvement of opioid analgesic system in verapamil modulation of pain. 

## Materials and Methods

**Animals.** Healthy adult male Wistar rats, weighing 250-280 g, were used in this study. Animals were maintained in groups of 6 per cage in a light-dark cycle (light on at 07:00 hr) at a controlled ambient temperature (22 ± 0.5 ˚C) with *ad libitum *access to food and water. Six rats were used for each experiment. All experiments were performed between 12:00 and 16:00. All research and animal care procedures were approved by Veterinary Ethics Committee of the Faculty of Veterinary Medicine of Urmia University and were performed in accordance with the National Institutes of Health Guide for Care and Use of Laboratory Animals. 

**Chemicals.** Chemicals used in the present study included verapamil hydrochloride, morphine sulfate and naloxone hydrochloride. Verapamil and naloxone hydrochloride were purchased from Sigma-Aldrich Chemical Co. (St. Louis, USA). Morphine sulfate was purchased from Temad Chemical Co. (Tehran, Iran). All drugs were dissolved in sterile normal saline 30 min before IP injections.

**Treatment groups.** The animals were divided into following groups of six rats each:

Group 1) This group received IP and intraplantar injections of normal saline; Group 2) This group received IP injection of normal saline before intraplantar injection of formalin; Groups 3, 4 and 5) In these groups, IP injection of verapamil at doses of 0.5, 2 and 8 mg kg^-1^, respectively, were performed before intraplantar injection of formalin; Groups 6, 7 and 8) In these groups, IP injection of morphine at doses of 1, 2 and 4 mg kg^-1^, respectively, were performed before intraplantar injection of formalin; Groups 9 and 10) In these groups, co-administrations of verapamil at doses of 0.5 and 1 mg kg^-1^ were performed with 1 mg kg^-1^ of morphine, respectively, before intraplantar injection of formalin; Groups 11 and 12) These groups received SC injection of naloxone (2 mg kg^-1^) with IP injection of morphine (2 mg kg^-1^) and verapamil (2 mg kg^-1^), respectively, before intraplantar injection of formalin.

The SC injection of naloxone and IP injections of verapamil and morphine were performed 35, 30 and 25 min before intraplantar injection of formalin, respectively. In the present study, the used doses of verapamil, morphine and naloxone were designed according to previous studies in which 1-30 mg kg^-1^ of verapamil, 2.5-10 mg kg^-1^ of morphine, and 1-2 mg kg^-1^ of naloxone have been used.^5^^,^^18^^-^^20^

**Orofacial pain.** Orofacial pain was induced according to the method described by Tamaddonfard *et al*.,^16^ and Erfanparast *et al*.^17^ Each rat was placed in plexiglass observation chamber (30 × 30 × 30 cm) with a mirror mounted at 45° beneath the floor to allow an unobstructed view of the orofacial region. After a 30-min adaptation period, 50 µL of 1.5% diluted formalin solution was subcutaneously (SC) injected into the left side of upper lip just lateral to the nose using a 29-gauge injection needle. Immediately following formalin injection, the rat was returned into the observation chamber. The time each animal spent face rubbing with ipsilateral forepaw was recorded (using a stopwatch), in consecutive 3-min blocks over a period of 45 min, and was considered as an index of nociception. Subcutaneous injection of formalin induced a stereotyped response characterized by two well distinct phases. In the present study, data collected between 0 and 3 min post-formalin injection represented the first (early) phase and data collected between 15 and 33 min after injection of formalin represented second (late) phase.^15^^-^^17^

**Statistical analysis.** Data obtained from 3 min blocks were analyzed by Excel (Version 2010; Microsoft, Redmond, USA) using factorial ANOVA followed by Duncanʼs test. Data obtained from the first and second phases were analyzed using one-way ANOVA followed by Duncanʼs test. In figures, all values are expressed as the mean ± SEM. A value of *p *< 0.05 was considered statistically significant.

## Results

**Fig. 1:**
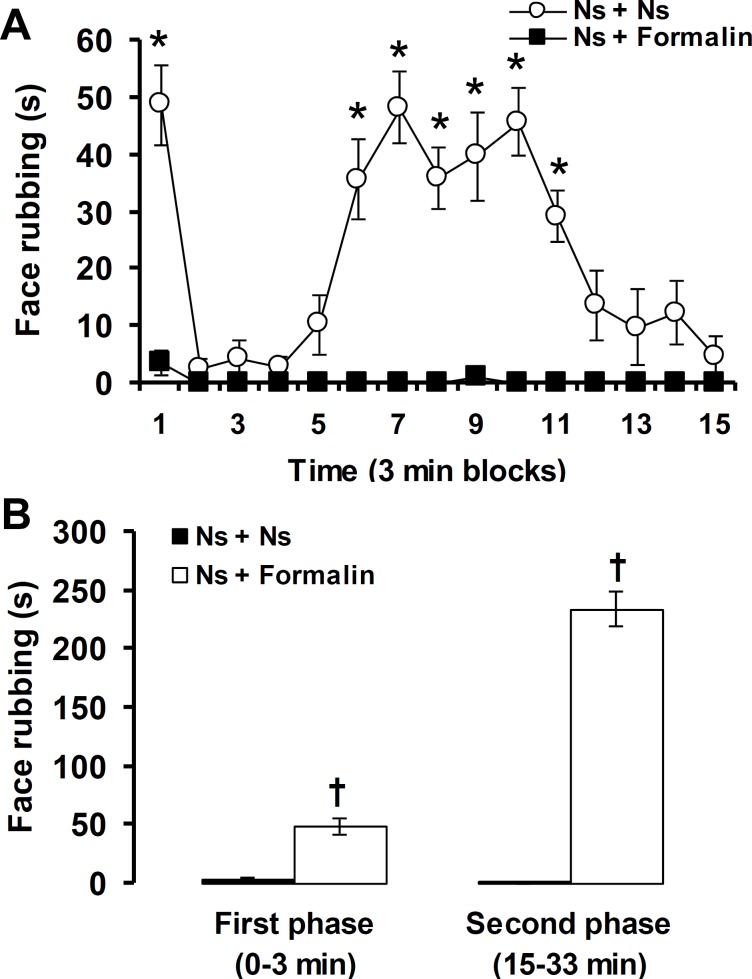
Three min blocks (A) and first and second phases (B) of pain response induced by injection of normal saline and formalin into upper lip. * *p* < 0.05 compared to Ns + Ns treated group and other three min blocks. † *p* < 0.05 compared to Ns + Ns treated group. Ns: Normal saline

Intraplantar injection of normal saline produced negligible pain responses in the first (first phase: 3.5 ± 2.2 sec) and ninth (second phase; 1.2 ± 1.0 sec) three min blocks. The SC injection of formalin into the upper lip region produced pain responses in the first and 6^th^ - 11^th^ three min blocks. Therefore, formalin produced a biphasic pattern (first phase: 0-3 min and second phase: 15-33 min) of pain response in orofacial region (Figs. 1A and 1B).

**Fig. 2:**
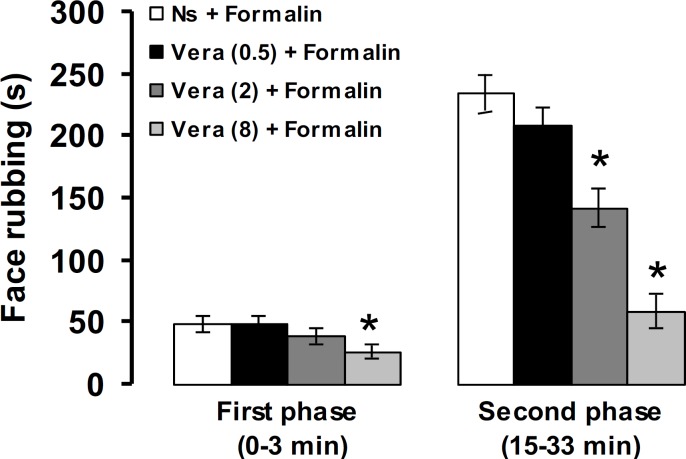
The effects of verapamil on formalin-induced orofacial pain response in rats. The numbers in the parenthesis show the doses of verapamil (mg) per kg of body weight. * *p* < 0.05 compared to Ns + Formalin treated group. Ns: Normal saline, Vera: Verapamil

Verapamil (0.5 mg kg^-1^, IP) produced no significant effects on the first and second phases of pain. The IP injection of verapamil (2 mg kg^-1^) significantly decreased the intensity of the second phase of pain (*p* < 0.05). The first and second phases of pain responses were significantly (*p *< 0.05) suppressed by IP injection of 8 mg kg^-1^ of verapamil (Fig. 2).

**Fig. 3:**
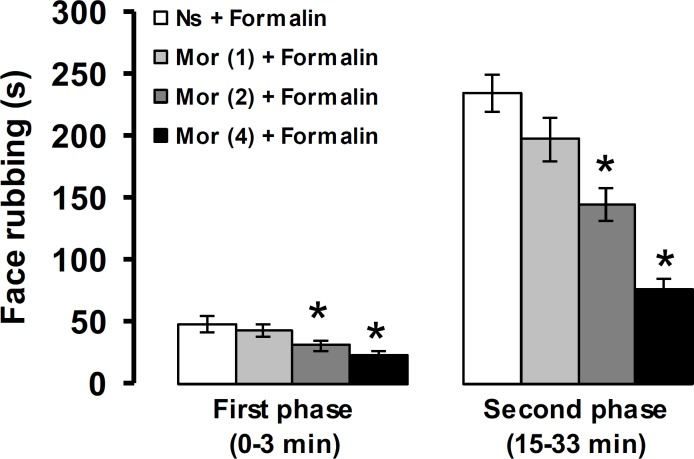
The effects of morphine on formalin-induced orofacial pain response in rats. The numbers in the parenthesis show the doses of morphine (mg) per kg of body weight. * *p* < 0.05 compared to Ns + Formalin treated group. Ns: Normal saline, Mor: Morphine

Figure 3 shows the effects of morphine on the first and second phases of pain response induced by formalin. The first and second phases of pain response were not altered by morphine at a dose of 1 mg kg^-1^. Morphine at doses of 2 and 4 mg kg^-1^ significantly suppressed the first and second phases of pain induced by formalin (*p* < 0.05). 

**Fig. 4:**
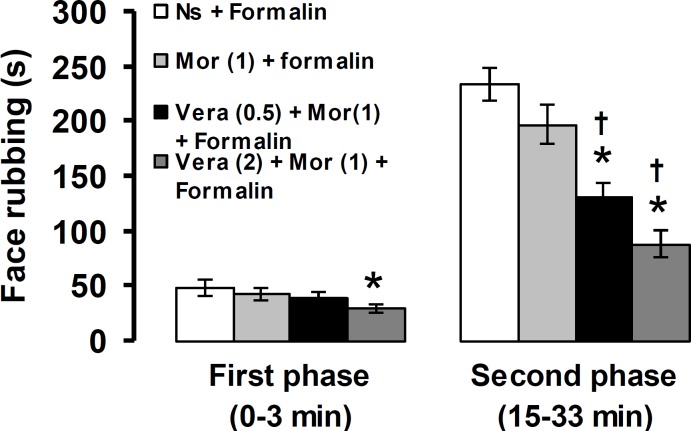
The effects of co-administration of verapamil and morphine on formalin-induced orofacial pain response. The numbers in the parenthesis show the doses of verapamil and morphine (mg) per kg of body weight. * *p* < 0.05 compared to Ns + Formalin treated group. † *p* < 0.05 compared to Mor (1) + Formalin treated group. Ns: Normal saline, Vera: Verapamil, Mor: Morphine

Figure 4 shows the effects of verapamil plus morphine on the first and second phase of pain induced by formalin. 

Co-administration of sub-analgesic doses of verapamil (0.5 mg kg^-1^) and morphine (1 mg kg^-1^) with no effect on the first phase, significantly reduced the second phase of formalin-induced pain (*p* < 0.05). The first and second phases of formalin pain responses were significantly decreased by co-administration of an analgesic dose of verapamil (2 mg kg^-1^) plus a sub-analgesic dose of morphine (1 mg kg^-1^), (*p* < 0.05).

**Fig. 5:**
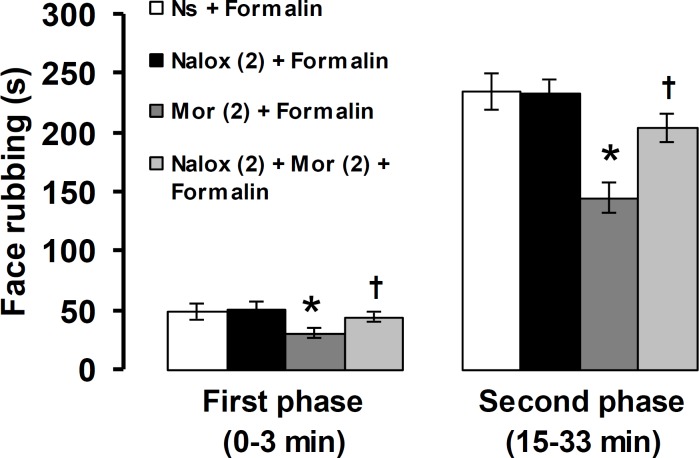
The effects of naloxone pretreatment on morphine-induced antinociception in orofacial pain response induced by formalin. The numbers in the parenthesis show the doses of naloxone and morphine (mg) per kg of body weight. * *p* < 0.05 compared to Ns + Formalin treated group. † *p* < 0.05 compared with to Mor (1) + Formalin treated group. Ns: Normal saline, Mor: Morphine, Nalox: Naloxone

Figure 5 shows the effects of naloxone alone and before morphine on the first and second phases of pain response induced by formalin. Naloxone (2 mg kg^-1^) alone did not change the intensity of the first and second phases of pain. Pretreatment with naloxone prior to morphine (2 mg kg^-1^) significantly prevented the first and second phase pain suppression induced by 2 mg kg^-1^ of morphine (*p* < 0.05). 

**Fig. 6:**
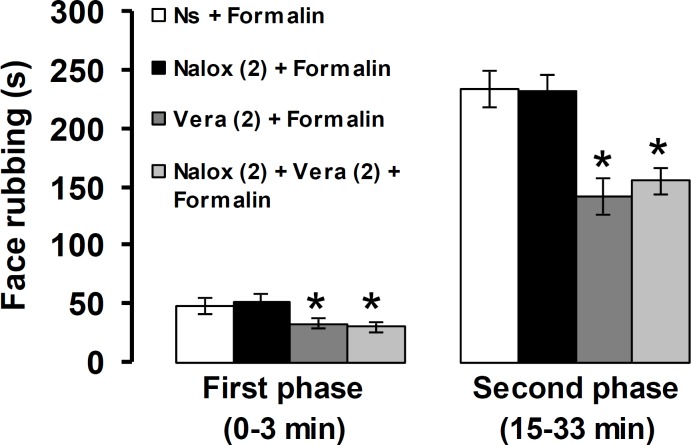
The effects of naloxone pretreatment on verapamil-induced antinociception in orofacial pain response induced by formalin. The numbers in the parenthesis show the doses of verapamil, naloxone and morphine (mg) per kg of body weight. * *p* < 0.05 compared to Ns + Formalin treated group. Ns: Normal saline, Vera: Verapamil, Nalox: Naloxone

Figure 6 shows the effects of naloxone before verapamil on the first and second phases of pain response induced by formalin. Pre-treatment with naloxone (2 mg kg^-1^) prior to verapamil (2 mg kg^-1^) did not prevent the antinociceptive effects induced by verapamil in the first and second phases of pain.

## Discussion

The present study shows that SC injection of 1.5% formalin into the upper lip produced a distinct biphasic (first phase: 0-3 min and second phase (15-33 min) pattern in the face rubbing performed by ipsilateral forepaw. The SC injection of formalin at the concentrations of 0.2-10% into the upper lip region induced a biphasic pattern of face rubbing in rats.^13^^-^^17^ During the orofacial formalin test, two distinct phases due to different mechanisms of nociception produces, the first phase is associated with direct stimulation of C-nociceptors, whereas the second phase is related to the release of inflammatory mediators such as prostaglandins.^14^ Face rubbing with the ipsilateral forepaw due to formalin injection into the upper lip, has been mentioned as a specific nociceptive response.^11^ Some researchers have reported vocalization, grooming and scratching due to electrical, mechanical, thermal and chemical (formalin) stimulation of the orofacial region in rats.^11^^-^^22^ Most of these studies have recorded the formalin-induced pain-related responses at three min blocks for a period of 45 min.^11^^-^^14^^,^^16^^-^^22^ The time schedule of five min blocks for a period of 1 hr for recording the formalin-induced orofacial pain have been also used.^15^^,^^23^

In the present study, IP injection of verapamil produced an antinociceptive effect by reducing formalin-induced face rubbing. Verapamil is a frequently prescribed calcium channel blocker used in treatment of hypertension, angina pectoralis and cardiac arrhythmias.^24^ The effect of verapamil on pain differs depending on dosage, route of administration and pain test used. The IP injection of verapamil had no effect on acetic acid-induced visceral and hot plate tests of nociception in mice,^25^^,^^26^ whereas in the formalin test in rats, verapamil produced an antinociceptive effect.^27^ Intrathecal administration of verapamil produced no effect on pain behaviors in neuropathic pain model derived from spinal nerve ligation.^28^ There is not any report showing the effects of verapamil on formalin-induced orofacial pain. Sukriti and Pandhi reported that IP injection of nimodipine, a LTCC blocker, attenuated facial grooming behavior induced by SC injection of formalin into the vibrissal pad in rats.^29^ These findings and the results of our study confirm the role of Ca^2+^ and its LTCCs in mediating inflammatory pain.

The results of the present study showed that morphine via a naloxone-sensitive mechanism reduced formalin-induced orofacial pain. Morphine acts through mu-opioid receptors, and naloxone is a competitive antagonist of mu-, kappa- and sigma-opioid receptors with higher affinity for the mu-opioid receptors.^30^ Morphine and naloxone have been frequently used to explore the role of endogenous opioid system in peripheral, spinal and supra-spinal trigeminal pain and analgesia mechanisms. Administration of morphine simultaneously with formalin reduced the early and late phases of formalin-induced facial pain in rats, and local injection of naloxone completely reversed the anti-nociceptive effect of morphine.^31^ Cervicomedullary intra-thecal injection of naloxone antagonized morphine-induced antinociception in the orofacial formalin test in rats.^32^ Microinjection of naloxone into the hippocampus prevented morphine-induced antinociception in formalin-induced orofacial pain in rats.^17^ However, naloxone had no effect on the intensity of orofacial pain induced by formalin, when microinjected into the hippocampus and dentate gyrus.^17^^,^^33^

Although, morphine is a gold standard analgesic commonly used to alleviate pain, some adverse effects such as constipation, respiratory depression, physical dependence and dizziness limit its use.^30^^,^^34^ Therefore, one might expect to eliminate these side effects by a strategy, which provides an effective treatment with a low dose of morphine. In the present study, an antinociceptive effect on the second phase of pain was observed when sub-analgesic doses of verapamil and morphine were co-administered. Both phases of formalin-induced pain were suppressed when an analgesic dose (2 mg kg^-1^) of verapamil co-administered with a sub-analgesic dose (1 mg kg^-1^) of morphine. It seems that a synergistic effect between verapamil and morphine is responsible for their pain suppressing effect in combination treatments. Intravenous injection of verapamil potentiated the analgesic effects of morphine on finger pressure and ice water immersion tests of nociception in humans.^35^ Pretreatment with verapamil potentiated the analgesic effects of morphine and increased serum levels of morphine in mice.^36^ Although pharmacological mechanisms for potentiating effect of verapamil on morphine antinociception are unclear, it is possible that verapamil enhances morphine-induced attenuation of nociceptive activity through blockade of Ca^2+^ channels. The present results also showed that verapamil via a naloxone-insensitive mechanism produced analgesia in the formalin-induced orofacial pain. It has been reported that naloxone affected neither both the inflammatory changes and nociceptive responses induced by formalin nor the anti-inflammatory and antinociceptive effects of nitrendipine, nicardipine, diltiazem and verapamil in rats.^37^ By considering the inhibitory effects of naloxone on opioid receptors,^30^^,^^34^ our findings indicated that endogenous opioid system was not involved in verapamil-induced antinociception.

In conclusion, the results of the present study showed antinociceptive effects of verapamil and morphine through suppressing both phases of formalin-induced orofacial pain. Co-administration of sub-analgesic doses of verapamil and morphine produced an antinociceptive effect only on second phase. Both phases of formalin-induced pain were suppressed when an analgesic dose of verapamil co-administered with a sub-analgesic dose of morphine. It seems that verapamil-induced antinociception was not mediated through endogenous opioid system.

## References

[1] Cox DH (2011). Ca2+-regulated ion channels. BMB Rep.

[2] Parekh AB, Putney JW Jr (2005). Store-operated calcium channels. Physiol Rev.

[3] Catteral WA, Prezereyes E, Snuth TP (2005). International union of pharmacology XLVIII Nomenclature and structure-function relationship of voltage-gated calcium channels. Pharmacol Rev.

[4] Dolphin AC (2009). Calcium channel diversity: Multiple roles of calcium channel subunits. Curr Opin Neurobiol.

[5] Prado WA (2001). Involvement of calcium in pain and anti-nociception. Braz J Med Biol Res.

[6] Park JF, Luo ZD (2010). Calcium channel functions in pain processing. Channels (Austin).

[7] Zamponi G, Lewis RJ, Todorovic SM (2009). Role of voltage-gated calcium channels in ascending pain pathways. Brain Res Rev.

[8] Amrutkar DV, Ploug KB, Olessen J (2011). Role for voltage calcium channels in calcitonin gene related peptide release in the rat trigeminovascular system. Neuro-science.

[9] Vijayan L, Bansal O, Ray IB (2012). Nimodipine down-regulate CGRP expression in the rat trigeminal nucleus caudalis. Indian J Exp Biol.

[10] Cheong E, Lee S, Choi BJ (2008). Tuning thalamic firing modes via simultaneous modulation of T- and L-type Ca2+ channels controls pain sensory gating in the thalamus. J Neurosci.

[11] Raboisson P, Dallel R (2004). The orofacial formalin test. Neurosci Biobehav Rev.

[12] Clavelou P, Pajot J, Dallel R (1989). Application of the formalin test to the study of orofacial pain in the rat. Neurosci Lett.

[13] Clavelou P, Dallel R, Orliaqguet T (1995). The orofacial formalin test in rats: Effects of different formalin concentrations. Pain.

[14] Dallel R, Raboisson P, Clavelou P (1995). Evidence for a peripheral origin of the tonic nociceptive response to subcutaneous formalin. Pain.

[15] Tamaddonfard E, Erfanparast A, Khalilzadeh E (2012). Effect of pilocarpine on the formalin-induced orofacial pain in rats. Vet Res Forum.

[16] Tamaddonfard E, Erfanparast A, Farshid AA (2011). Interaction between histamine and morphine at the level of the hippocampus in the formalin-induced orofacial pain in rats. Pharmacol Rep.

[17] Erfanparast A, Tamaddonfard E, Farshid AA (2010). Antinociceptive effect of morphine microinjections into the dorsal hippocampus in the formalin-induced orofacial pain in rats. Vet Res Forum.

[18] Quintans-Junior LJ, Melo MS, De Sousa DP (2010). Antinociceptive effect of citronellal in formalin-, capsicin-, and glutamate-induced orofacial nociception in rodents and its action on nerve excitability. J Orofac Pain.

[19] Burgos E, Pascual D, Martin MI (2010). Antinociceptive effect of the cannabinoid agonist, WIN 55, 212-2, in the orofacial and temporomandibular formalin tests. Eur J Pain.

[20] Santana MF, Quintans-Junior LJ, Socrates C (2011). p-Cymene reduces orofacial niciceptive response in mice. Rev Bras Farmacogn.

[21] Sugiyo S, Uehashi D, Satoh F (2009). Effects of systemic bicuculline or morphine on formalin-evoked pain-related behaviour and c-Fos expression in trigeminal nuclei after formalin injection into the lip or tongue in rats. Exp Brain Res.

[22] Ro JY, Capra N, Masri R (2003). Development of a behavioral assessment of craniofacial muscle pain in lightly anesthetized rats. Pain.

[23] Zeredo JL, Sasaki KM, Takeuchi Y (2005). Antinociceptive effect of Er:YAG laser irradiation in the orofacial formalin test. Brain Res.

[24] Das R, Plow EF (2010). A new function for old drugs. Cell Cycle.

[25] Quijada L, Germany A, Hernández (1992). Effects of calcium channel antagonists and Bay K 8644 on the analgesic response to pentazocine and U 50488H. Gen Pharmacol.

[26] EL-Azab MF, Moustafa YM (2012). Influence of calcium channel blockers on anticonvulsant and antinociceptive activities of valporic acid in pentylenetetrazole-kindled mice. Pharmacol Rep.

[27] Miranda HF, Bustamante D, Kramer V (1992). Anti- nociceptive effects of Ca2+ channel blockers. Eur J Pharmacol.

[28] Chaplan SR, Pogrel JW, Yaksh TL (1994). Role of voltage-dependent calcium channel sutypes in experimental tactile allodynia. J Pharmacol Exp Ther.

[29] Sukriti DH, Pandhi P (2004). Potentiation of antihyperalgesic activity of diclofenac by nimodipine in a formalin model of facial pain in rats. Methods Find Exp Clin Pharmacol.

[30] Trescot AM, Datta S, Lee M (2008). Opioid Pharmacology. Pain Physician.

[31] Eisenberg E, Vos BP, Strassman AM (1996). The peripheral antinociceptive effect of morphine in a rat model of facial pain. Neuroscience.

[32] Grabow TS, Dougherty PM (2001). Cervicomedullary intrathecal injection of morphine produces antinociception in the orofacial formalin test in the rat. Anesthesiology.

[33] Khalilzadeh E, Tamaddonfard E, Farshid AA (2010). Thioperamide-induced antinociception is mediated through endogenous opioid system in the dentate gyrus of adult rats. Vet Res Forum.

[34] Benyamin R, Trescot AM, Datta S (2008). Opioid complications and side effects. Pain Physician.

[35] Vaupel DB, Lange WR, London ED (1993). Effects of verapamil on morphine-induced euphoria, analgesia and respiratory depression in humans. J Pharmacol Exp Ther.

[36] Shimizu N, Kishioka S, Maeda T (2004). Role of pharmacokinetic effects in the potentiation of morphine analgesia by L-type calcium channel blockers in mice. J Pharmacol Sci.

[37] Gurdal H, Sara Y, Tulunay FC (1992). Effects of calcium channel blockers on formalin-induced nociception and inflammation in rats. Pharmacology.

